# Difficult-to-treat, complex-to-manage, treatment-refractory spondyloarthritis: semantics or substance?

**DOI:** 10.1097/BOR.0000000000001173

**Published:** 2026-06-16

**Authors:** Olivier Fakih, Stephanie Rose Harrison, Helena Marzo-Ortega

**Affiliations:** a NIHR Leeds Biomedical Research Centre, The Leeds Teaching Hospitals NHS Trust and Leeds Institute of Rheumatic and Musculoskeletal Medicine, University of Leeds, Leeds, UK; b Université Marie et Louis Pasteur, Service de rhumatologie, CHU de Besançon, Besançon, France

**Keywords:** axial spondyloarthritis, difficult-to-manage, difficult-to-treat, psoriatic arthritis, treatment-refractory

## Abstract

**Purpose of review:**

The concept of difficult-to-treat (D2T) disease is increasingly recognized across immune-mediated inflammatory diseases, and was recently introduced in spondyloarthritis (SpA). Several terms, including difficult-to-manage (D2M), complex-to-manage (C2M), and treatment-refractory (TR) describe disease states characterized by persistent symptoms and inadequate response to targeted therapies. This review aims to clarify the emerging constructs of D2M and TR disease in axial spondyloarthritis (axSpA) and psoriatic arthritis (PsA), and their implications for emerging research in this area.

**Recent findings:**

Recent initiatives from ASAS, EULAR, and GRAPPA have proposed definitions to frame D2T clinical scenarios in axSpA and PsA. These converge on distinguishing treatment-refractory disease, characterized by persistent objective inflammation despite multiple targeted therapies, from broader D2M/C2M states driven by multifactorial contributors including non-inflammatory factors. Emerging data suggest that clinical and biological heterogeneity across disease domains may contribute to these phenotypes. Challenging phenotypic presentations often display overlapping features between axSpA and PsA, supporting the concept of a continuum across the SpA spectrum.

**Summary:**

Distinguishing TR disease from broader D2M/C2M states is essential for avoiding inappropriate treatment escalation, and supporting personalized multidisciplinary care. Further research is needed to validate these definitions, determine contributing factors and their prevalence, and clarify the molecular mechanisms underlying treatment refractory disease.

## INTRODUCTION

The conceptual understanding of difficult-to-treat (D2T) disease is rapidly evolving as a clinical and research need in immune-mediated diseases (IMIDs), including spondyloarthritis (SpA). Several terms are used in the literature, including “difficult-to-manage” (D2M), “complex-to-manage” (C2M), “treatment-resistant” and “treatment-refractory” (TR) [1^▪▪^,^2^^▪▪^,^3^^▪▪^] aiming to encompass the nuance of these disease states [4^▪^]. More than a terminological exercise, the current challenge is to determine the interchangeability of these terms and whether the underpinning constructs identify persistent objective inflammation, persistent symptoms driven by other, non-inflammatory, mechanisms, or overlapping phenotypes across the SpA spectrum, before fully understanding their potential clinical applicability. 

**Box 1:**
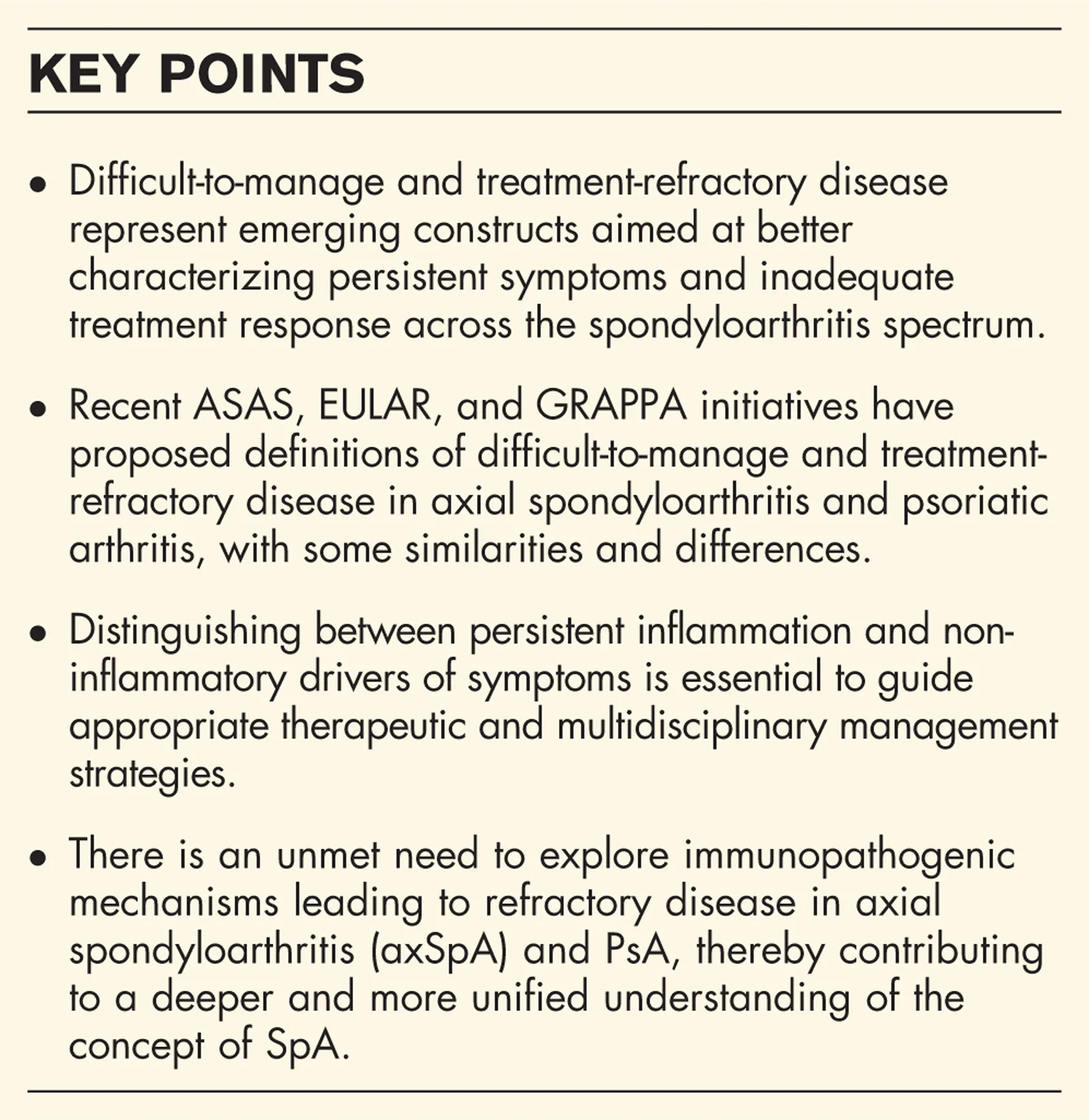
no caption available

## WHY WE NEED A DIFFICULT-TO-TREAT CONSTRUCT IN SPONDYLOARTHRITIS

The treatment of IMIDs has been transformed in the last 20 years with the rapid introduction of advanced therapies with multiple mechanisms of action aimed at specific biologic targets. In parallel, the instigation of treat-to-target strategies has contributed to more effective control of inflammation resulting in optimized outcomes for affected individuals with current treatment guidelines advocating for tight inflammatory control as the primary goal for maximizing health-related quality of life. However, at the bedside, challenges remain due to the inability to personalize treatment prescription due to a lack of biomarkers of treatment response, leading to a trial-and-error approach.

In the context of psoriatic arthritis (PsA) and axial spondyloarthritis (axSpA), the representative SpA phenotypes, the multiplicity of tissue involvement and added comorbidities make treatment decisions challenging with the main drivers being clinical phenotype and cost. The expanding therapeutic arsenal for PsA [5] and axSpA [6] presents real opportunities for patients who have suboptimal responses to first-line treatment. Approximately 30% of patients with PsA do not respond adequately to the first biologic disease-modifying antirheumatic drug (bDMARD), and drug survival tends to decrease with every subsequent bDMARD [7,8], highlighting the importance of knowing which drug to switch to and when. If a treatment is stopped and then restarted, there is no guarantee that the same efficacy of response will be achieved; hence, the consequences of an unsuccessful treatment switch are profound. For people living with these diseases, lack of treatment response also referred to as “treatment failure” means loss of disease control, which potentially has a significant impact on their health, physical, personal, and financial well being. Additionally, treatment failure can lead to suboptimal clinical outcomes, drug waste, and increased health service utilization and bears broader socioeconomic costs.

In SpA, the collision of diverse disease expression and symptoms occurs often through the aggregation of multiple tissue involvement and the high prevalence of inflammatory and non-inflammatory comorbidity, exacerbating the disease burden and prognosis. In addition, external drivers including social, environmental, educational or psychological factors may interact, leading to more complex health outcomes and affecting treatment response. Ultimately, as opposed to rheumatoid arthritis (RA), defining one overarching concept for D2T disease in SpA can be challenging. Indeed, D2T disease can be reasonably construed to mean different things to care providers and patients; as highlighted in recent surveys of expert and patients’ opinion on the scope of the D2T PsA concept [9–11].

## BEYOND SEMANTICS – WHY TERMINOLOGY MATTERS

When exploring D2T disease in SpA, two conceptual aspects are key. First, it is essential to address the multifaceted nature of these conditions, including multidomain/tissue involvement, and high morbidity burden including psychosocial factors. Second, it is paramount to distinguish between non-inflammatory and inflammatory clinical scenarios. Dissecting these concepts allows for full implementation of comprehensive targeted and holistic management approaches.

Indeed, clinical symptoms suggestive of treatment nonresponse can be impacted by co-existing secondary osteoarthritis, cardiometabolic disease, poor mental health, and chronic pain syndromes, among other factors, which often require a holistic and nonpharmacological intervention with input from a multidisciplinary team rather than a change in b/tsDMARD therapy. These scenarios need to be differentiated from those contributing to ongoing inflammation, which can be objectively assessed using serum or imaging markers. The latter, if persistent despite pharmacological intervention with multiple advanced therapies, may be referred to as treatment-refractory and represents a truly difficult-to-treat clinical scenario which may be ongoing for years. On the other hand, patients with other drivers of persistent symptoms, for instance drug intolerability, high infection risk or poor compliance would be classified as having difficult-to-manage disease at a certain moment and time, although a satisfactory treatment response may eventually be achieved by a successful intervention for example a change of drug, administering device or an alternative dosing schedule as agreed with the patient. Thus, differentiating those D2T scenarios which may require intervention to address ongoing biological inflammation from those where nonbiological factors may be contributing to ongoing symptomatology is key, and represents a significant advance from one single overarching definition, as proposed for RA [12].

In RA, the distinction between persistent inflammatory and non-inflammatory mechanisms in refractory disease was introduced later with the concepts of persistent inflammatory and non-inflammatory mechanisms in refractory rheumatoid arthritis (PIRRA and NIRRA) proposed [13], specifying that these were not mutually exclusive. By contrast, this distinction was incorporated from the outset within the proposed frameworks for axSpA and PsA and incorporated within the concepts of D2M/C2M disease, encompassing multiple drivers of persistent symptoms, and TR disease, which represents a subset of D2M/C2M characterized by objective evidence of ongoing inflammation and the exclusion of alternative non-inflammatory drivers of symptoms.

## CLINICAL AND BIOLOGICAL HETEROGENEITY BEHIND D2M AND TR PHENOTYPES

Part of this distinction between D2M and TR phenotypes may also reflect genuine biological heterogeneity across disease domains. Co-existing extra-musculo-skeletal manifestations, such as psoriasis, uveitis, or inflammatory bowel disease, may complicate treatment options and contribute to D2M or TR disease, such as the contraindication of interleukin (IL)-17 inhibitors in patients with active inflammatory bowel disease (IBD). The immunological and dynamic heterogeneity observed across different clinical domains in SpA provides insights into the development of D2M or TR phenotypes. For example, in PsA, IL-23 and IL-17 inhibitors show high efficacy in cutaneous disease but have more modest effects on synovitis or enthesitis. In axSpA, IL-17 inhibitors are effective for spinal disease, whereas IL-23 inhibitors have not shown efficacy in this domain [14]. However, the entheseal bone with predominantly osteitis driving axSpA and the inflamed soft tissues primarily involved in PsA have distinct immune cell and stromal compositions, which may explain the differential response to immunomodulatory therapy, especially IL-23 inhibition [15]. In general, the assessment of treatment nonresponse in PsA is both clinically challenging and dynamic, with patients moving in and out of a state of nonresponse in different disease domains at different time points.

## CURRENT DEFINITIONS OF D2M AND TR AXSp-A AND PsA

Expert consensus definitions have been recently proposed almost contemporarily aiming to frame these constructs in axSpA by the Assessment for Spondyloarthritis International Society (ASAS) [1^▪▪^] and PsA by the European Alliance of Associations for Rheumatology (EULAR) [2^▪▪^] and the Group for Research and Assessments of Psoriasis and Psoriatic Arthritis (GRAPPA) [3^▪▪^]. Although methodologies varied between each project (Table 1), these expert groups aligned on the need to differentiate two stand-alone constructs encompassed under the overarching difficult-to-treat umbrella: difficult-to-manage covering the wider range of biological and nonbiological factors contributing to treatment nonresponse and treatment-refractory disease, characterized by objective evidence of biological inflammation. This is a key differentiator from the initial D2T proposals in RA [12] and other diseases.

**Table 1:** Comparison between the ASAS difficult-to-manage axial spondyloarthritis (D2M-axSpA), EULAR difficult-to-manage psoriatic arthritis (D2M-PsA), and GRAPPA complicate to-manage psoriatic arthritis (C2M-PsA) and treatment-refractory (TR) definitions

	D2M and TR-axSpA (ASAS)	D2M and TR-PsA (EULAR)	C2M and TR-PsA (GRAPPA)	
Literature review strategy	Scoping review s Set by the steering committee then later presented to the wider TF	3-research question SLR Set by the steering committee based on scope/aims agreed at the 1st TF meeting	Scoping review Set by the steering committee then later presented to the wider TF	
Supporting surveys	None	One international survey (healthcare professionals) Conducted prior to SLR	Two international surveys (healthcare professionals and patients) Conducted prior to SLR	
Formulation and refining of the definition	Formulation by the TF Input from patient representatives	Formulation by steering committee only Input from patient representatives	Formulation by the TF Input from patient representatives and pharmaceutical industry	
Validation of the definition	Consensus-based definition (2 Delphi rounds)	Consensus-based definition (EULAR SOPs)	Consensus-based definition (2 Delphi rounds)	
D2M or C2M definition	Therapeutic failures	Inadequate response to ≥2 b/tsDMARDs with ≥2 MoA	Inadequate response to ≥2 b/tsDMARDs with ≥2 MoA	Inadequate response to ≥1 b/tsDMARDs
	Signs of active disease	At least 1 of: - High or very high disease activity - Signs or symptoms suggestive of active disease - Rapid radiographic spinal progression - Reduction in quality of life	At least 1 of: - Failure to achieve or maintain LDA - Objective evidence of inflammation - Active EMMs - Other drivers: comorbidities, psychosocial, etc.	Persistent PsA-related symptoms
	Subjective perception	Symptoms considered as problematic by patient and/or rheumatologist	Management perceived as problematic by patient and/or rheumatologist	Symptoms considered as problematic by patient and/or rheumatologist
TR definition	D2M-axSpA and: - Objective evidence of inflammation (CRP+ or MRI+) - Other drivers excluded - High disease activity (ASDAS ≥2.1)	D2M-PsA and: - Objective evidence of inflammation - Other drivers excluded - Failure to achieve or maintain LDA OR active EMMs	C2M-PsA and: -Objective evidence of inflammation - Other drivers excluded - Inadequate response to ≥3 PsA therapies including ≥2 b/tsDMARDs with ≥2 MoA - Symptoms considered as problematic by patient AND rheumatologist	
Temporal element	No	No	No	
Drug intolerance/ contraindication/ side effects considered	Yes	Yes	Yes	

EMMs, extra-musculo-skeletal manifestations, LDA, low disease activity, MoA, mode of action, SLR, systematic literature review, SOPs, standard operating procedures, TF, task force.

However, differences exist in the conceptual approach behind each construct's framework. The ASAS axSpA and EULAR PsA definitions are aligned in the utilization of stricter entry thresholds aimed to capture the complexity of phenotypic and systemic manifestations of disease, whereas the GRAPPA PsA proposal utilizes a broader entry criterion reflected in new nomenclature (“complex-to-manage”). Encouragingly, many similarities are found between the different definitions, with wide appreciation of subsets of PsA that could be further defined based on objective markers of inflammation and ongoing disease activity. Clearly, the lack of background data makes this type of work more challenging as reflected on the different weighting of patients and physician perspectives used in the definitions, and on the number of b/tsDMARD failures required to characterize a D2M/C2M or “treatment refractory” state. Patient representatives and clinical experts contributing to the EULAR task force for example, were in agreement that considering failure to respond to one single drug or mechanism of action did not constitute a difficult to manage scenario, just “real life” since a high proportion of patients, particularly female, do not achieve low disease activity states or full remission of symptoms and signs over the course of their disease. In addition, the reasons behind treatment “failure” need to be fully explored at the individual level,  as these will be different in everyone, with lack of compliance (of multiple origin) being a main contributor. Importantly, these definitions reflect a dynamic state that may fluctuate over time, with further work needed to establish whether a temporal framework may be feasibly incorporated.

Prospective studies are now needed to fully evaluate the performance of these definitions in the research field to standardize populations. This is key to avoid misuse of the definitions in routine clinical setting, and before their clinical utility is confirmed, including possible applicability in healthcare systems that treat different ethnicities and have differing access to resources such as MRI or genetic testing.

## THE CONSTRUCT OF D2M AND TR DISEASE AS ARGUMENT FOR A UNIFYING SpA DISEASE SPECTRUM

**FIGURE 1:**
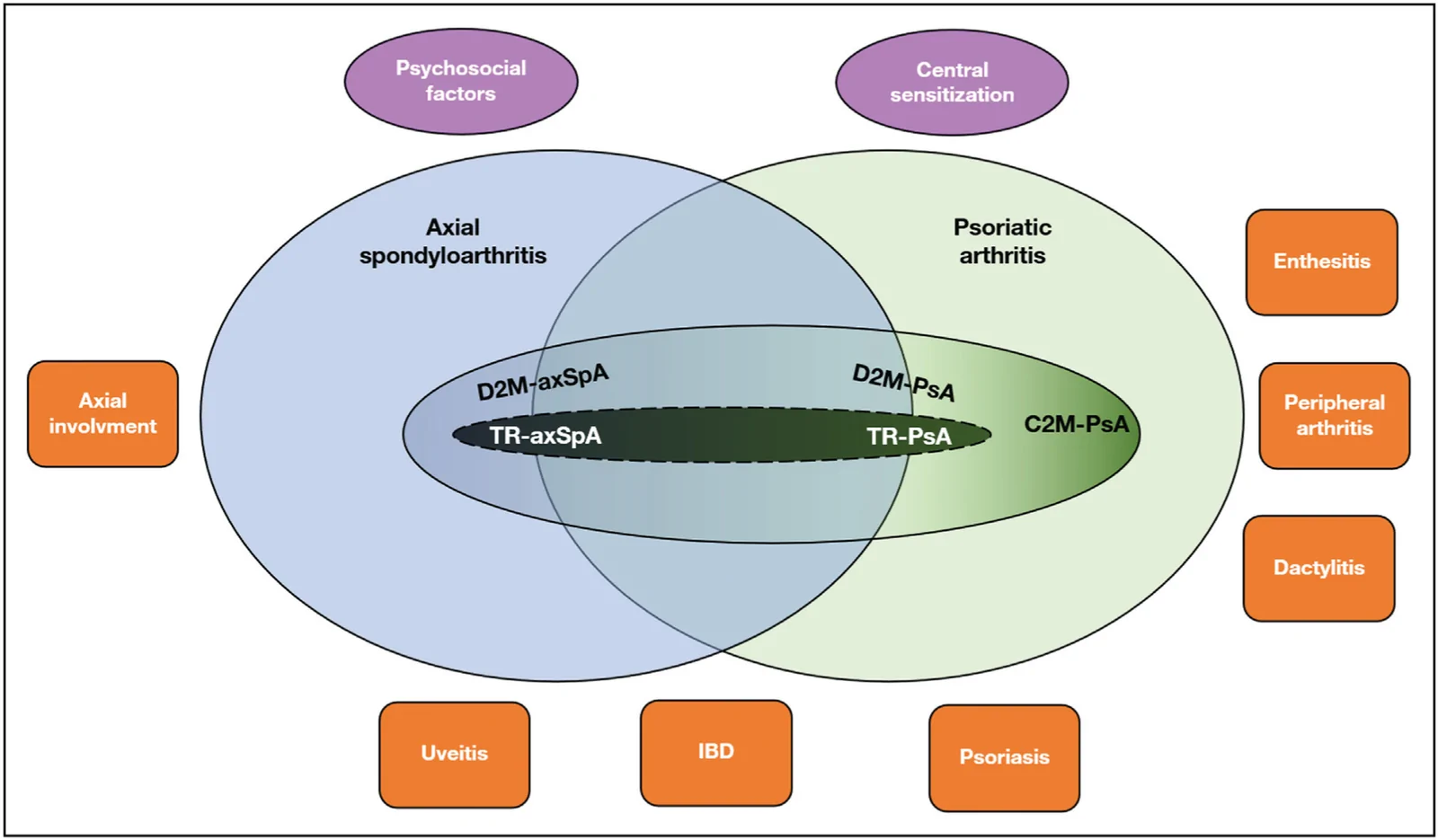
Management constructs (D2M/C2M and TR) across the SpA spectrum, and their relationship with transversal clinical domains and contextual drivers. The two overlapping circles represent predominantly axial spondyloarthritis (axSpA) and psoriatic arthritis (PsA) disease phenotypes. The central shaded area depicts difficult-to-manage (D2M) disease as an umbrella concept, within which difficult-to-manage axial SpA (D2M-axSpA) and difficult-to-manage/complex-to-manage PsA (D2M/C2M-PsA) are positioned – C2M-PsA being wider than D2M-PsA, and treatment-refractory subsets (TR-axSpA and TR-PsA) are shown as narrower constructs within D2M/C2M, representative of a smaller group. External boxes illustrate transversal elements that may occur anywhere across the SpA spectrum: orange boxes represent key clinical inflammatory manifestations, while purple ellipses represent contextual/non-inflammatory drivers that may contribute to symptom burden and perceived nonresponse. IBD, Inflammatory bowel disease.

Overall, the heterogeneity of these challenging clinical scenarios becomes even more apparent when difficult-to-manage and treatment-refractory phenotypes are examined across the broader SpA spectrum. A key finding of the recent literature was the report, in D2M axSpA cohorts, of peripheral joint involvement and psoriasis [16,17^▪^,^18^], which could classify these patients as having PsA. Furthermore, in the context of PsA, axial involvement, which may classify some patients as having axSpA, was noted in some PsA studies reporting difficult-to-manage clinic scenarios [19^▪^]. Indeed, earlier studies reporting on D2T SpA populations described this mixed phenotype with highly inflammatory, progressive and erosive peripheral and axial manifestations requiring novel therapeutic interventions such as IL-6 inhibition or biologic combination therapy [20,21]. This phenotypic convergence in complex scenarios of D2M axSpA and PsA warrants careful consideration, as it challenges the conventional nosological distinction between the two clinical entities while reinforcing the concept of a phenotypic continuum within the SpA disease spectrum [22] (Fig. 1). A more systematic investigation of these overlaps could reveal whether the current classification systems inadvertently obscure shared pathogenic pathways or clinical domains that are more relevant to therapeutic decisions. Ultimately, embracing the complexity of SpA phenotypes, particularly in clinical scenarios considered to have TR disease, will lead to more individualized and effective management strategies.

The studies published to date remain few and are widely heterogeneous in terms of the patients included and the methods used to apply definitions. Nevertheless, the high frequency of certain characteristics associated with D2M, including TR status, shows a certain consistency, notably female sex and high baseline disease activity. Clearly, our current understanding represents an evolving framework. Going forward, studies are needed to validate the robustness of the recently proposed definitions of D2M/C2M and TR axSpA [1^▪▪^] and PsA [2^▪▪^,^3^^▪▪^], establish their true prevalence, and to explore the factors more likely to be associated with these disease states.

## CONCLUSION

In conclusion, there is increasing awareness of the need to dissect the factors contributing to D2M and TR disease in SpA, with new definitions expected to help direct research in this area. The clinical scenarios of TR disease in axSpA and PsA cohorts represent a challenge at the clinical level, requiring a paradigm shift in their therapeutic approach. In this light, the emerging literature underscores the need to explore immunopathogenic mechanisms leading to treatment refractory disease, thereby contributing to a deeper understanding of the concept of SpA.

## Acknowledgements

*H.M.O. is supported by the NIHR Leeds Biomedical Research Centre (LBRC), The Leeds Teaching Hospitals National Health Service (NHS) Trust and is in receipt of an NIHR Senior Clinical and Practitioner Research Award (NIHR304573). The views expressed are those of the authors and not necessarily those of the (UK) NHS, the NIHR, or the (UK) Department of Health.*

*Author contributions: O.F., S.R.H. and H.M.O. conceptualized the manuscript. O.F. and S.R.H. wrote the first draft of the manuscript with support from H.M.O. All authors contributed to manuscript writing and read and approved the final version of the manuscript.*

### Financial support and sponsorship

*S.R.H. received speaker fees for a nonpromotional educational talk from Janssen, UCB and Novartis. She has received support to attend conferences from UCB and Janssen.*

*H.M.O. has received institutional research support from Janssen, Novartis, Pfizer and UCB and consultancy honoraria and/or speaker fees from AbbVie, Advanz Pharma, Amgen, Biogen, Eli-Lilly, Janssen, Moonlake, Novartis, Pfizer, Takeda, UCB and Zuellig-Pharma.*

*Funding: No funding was awarded for this work.*

### Conflicts of interest

*O.F. has received consultancy honoraria and speaker fees from U.C.B.*

## REFERENCES AND RECOMMENDED READING

Papers of particular interest, published within the annual period of review, have been highlighted as:

▪ of special interest
▪▪ of outstanding interest

## References

[1▪▪] Poddubnyy D Navarro-Compán V Torgutalp M. The Assessment of SpondyloArthritis International Society (ASAS) consensus-based expert definition of difficult-to-manage, including treatment-refractory, axial spondyloarthritis. Ann Rheum Dis 2025; 84:538–546. 39955166 10.1016/j.ard.2025.01.035

[2▪▪] Marzo-Ortega H Harrison SR Fragoulis GE. EULAR points to consider and consensus definitions for difficult-to-manage and treatment-refractory psoriatic arthritis. Ann Rheum Dis 2025; 85:61–74. 41168056 10.1016/j.ard.2025.10.002

[3▪▪] Proft F Ribeiro AL Singla S. Consensus definitions of complex-to-manage and treatment-refractory psoriatic arthritis: a GRAPPA initiative. Nat Rev Rheumatol 2026; 22:132–144. 41310208 10.1038/s41584-025-01329-3

[4▪] Singla S Ribeiro A Torgutalp M. Difficult-to-treat psoriatic arthritis (D2T PsA): a scoping literature review informing a GRAPPA research project. RMD Open 2024; 10:e003809. 38191215 10.1136/rmdopen-2023-003809 PMC10806599

[5] Gossec L Kerschbaumer A Ferreira RJO. EULAR recommendations for the management of psoriatic arthritis with pharmacological therapies: 2023 update. Ann Rheum Dis 2024; 83:706–719. 38499325 10.1136/ard-2024-225531 PMC11103320

[6] Ramiro S Nikiphorou E Sepriano A. ASAS-EULAR recommendations for the management of axial spondyloarthritis: 2022 update. Ann Rheum Dis 2023; 82:19–34. 36270658 10.1136/ard-2022-223296

[7] Merola JF Lockshin B Mody EA. Switching biologics in the treatment of psoriatic arthritis. Semin Arthritis Rheum 2017; 47:29–37. 28363434 10.1016/j.semarthrit.2017.02.001

[8] Haddad A Stein N Feldhamer I. Biologic switching in psoriatic arthritis: Insights from real-world data and key risk factors. Semin Arthritis Rheum 2025; 73:152737. 40267668 10.1016/j.semarthrit.2025.152737

[9] Ribeiro AL Singla S Chandran V. Deciphering difficult-to-treat psoriatic arthritis (D2T-PsA): a GRAPPA perspective from an international survey of healthcare professionals. Rheumatol Adv Pract 2024; 8:rkae074. 38912423 10.1093/rap/rkae074 PMC11193309

[10] Ribeiro AL Singla S Hay-Rollins C. Deciphering difficult-to-treat psoriatic arthritis: insights from an international survey of patients with psoriatic arthritis. Rheumatology 2025; 64:4641–4649. 40221862 10.1093/rheumatology/keaf207

[11] Marzo-Ortega H Harrison SR Nagy G. Time to address the challenge of difficult to treat psoriatic arthritis: results from an international survey. Ann Rheum Dis 2024; 83:403–404. 37963707 10.1136/ard-2023-225087

[12] Nagy G Roodenrijs NM Welsing PM. EULAR definition of difficult-to-treat rheumatoid arthritis. Ann Rheum Dis 2021; 80:31–35. 33004335 10.1136/annrheumdis-2020-217344 PMC7788062

[13] Buch MH Eyre S McGonagle D. Persistent inflammatory and non-inflammatory mechanisms in refractory rheumatoid arthritis. Nat Rev Rheumatol 2021; 17:17–33. 33293696 10.1038/s41584-020-00541-7

[14] Baeten D Østergaard M Wei JC-C. Risankizumab, an IL-23 inhibitor, for ankylosing spondylitis: results of a randomised, double-blind, placebo-controlled, proof-of-concept, dose-finding phase 2 study. Ann Rheum Dis 2018; 77:1295–1302. 29945918 10.1136/annrheumdis-2018-213328 PMC6104676

[15] McGonagle D David P Macleod T. Predominant ligament-centric soft-tissue involvement differentiates axial psoriatic arthritis from ankylosing spondylitis. Nat Rev Rheumatol 2023; 19:818–827. 37919337 10.1038/s41584-023-01038-9

[16] Smits ML Webers C Vonkeman HE. Exploring difficult-to-manage axial spondyloarthritis: results from a Dutch clinical practice registry. Rheumatology 2025; 64:3816–3825. 40036773 10.1093/rheumatology/keaf120 PMC12107025

[17▪] Proft F Lembke S Weiß A. Frequency of difficult-to-manage and treatment-refractory axial SpA: insights from the German RABBIT-SpA register using recent ASAS definitions. Rheumatology 2026; 65:keaf641. 41335412 10.1093/rheumatology/keaf641 PMC12822491

[18] Kougkas N Tsafis K Deligeorgakis D. POS0121 difficult-to-manage patients with axial spondyloarthritis: insights from the Greek AxSpA Registry. Ann Rheum Dis 2025; 84:415.

[19▪] Vassilakis KD Papagoras C Fytanidis N. Identification and characteristics of patients with potential difficult-to-treat psoriatic arthritis: exploratory analyses of the Greek PsA registry. Rheumatology 2024; 63:2427–2432. 38759119 10.1093/rheumatology/keae263 PMC11371370

[20] De Marco G McGonagle D Mathieson HR. Combined inhibition of tumour necrosis factor-alpha and interleukin-12/23 for long-standing, refractory psoriatic disease: a differential role for cytokine pathways? Rheumatology 2018; 57:2053–2055. 29982709 10.1093/rheumatology/key199

[21] Merashli M De Marco G Podgorski M. Evidence of response to IL-6 inhibition in some cases of refractory spondyloarthritis-associated peripheral synovitis. Ann Rheum Dis 2016; 75:1418–1420. 27059696 10.1136/annrheumdis-2016-209275

[22] Michelena X Sepriano A Zhao SS. Exploring the unifying concept of spondyloarthritis: a latent class analysis of the REGISPONSER registry. Rheumatology 2024; 63:3098–3105. 38237920 10.1093/rheumatology/keae005

